# Alternative Splicing in Neurogenesis and Brain Development

**DOI:** 10.3389/fmolb.2018.00012

**Published:** 2018-02-12

**Authors:** Chun-Hao Su, Dhananjaya D, Woan-Yuh Tarn

**Affiliations:** ^1^Institute of Biomedical Sciences, Academia Sinica, Taipei, Taiwan; ^2^Taiwan International Graduate Program in Molecular Medicine, National Yang-Ming University and Academia Sinica, Taipei, Taiwan

**Keywords:** alternative splicing, splicing factors, neurogenesis, neuronal differentiation, neuronal migration, neuronal development

## Abstract

Alternative splicing of precursor mRNA is an important mechanism that increases transcriptomic and proteomic diversity and also post-transcriptionally regulates mRNA levels. Alternative splicing occurs at high frequency in brain tissues and contributes to every step of nervous system development, including cell-fate decisions, neuronal migration, axon guidance, and synaptogenesis. Genetic manipulation and RNA sequencing have provided insights into the molecular mechanisms underlying the effects of alternative splicing in stem cell self-renewal and neuronal fate specification. Timely expression and perhaps post-translational modification of neuron-specific splicing regulators play important roles in neuronal development. Alternative splicing of many key transcription regulators or epigenetic factors reprograms the transcriptome and hence contributes to stem cell fate determination. During neuronal differentiation, alternative splicing also modulates signaling activity, centriolar dynamics, and metabolic pathways. Moreover, alternative splicing impacts cortical lamination and neuronal development and function. In this review, we focus on recent progress toward understanding the contributions of alternative splicing to neurogenesis and brain development, which has shed light on how splicing defects may cause brain disorders and diseases.

## Introduction

**Figure 1:**
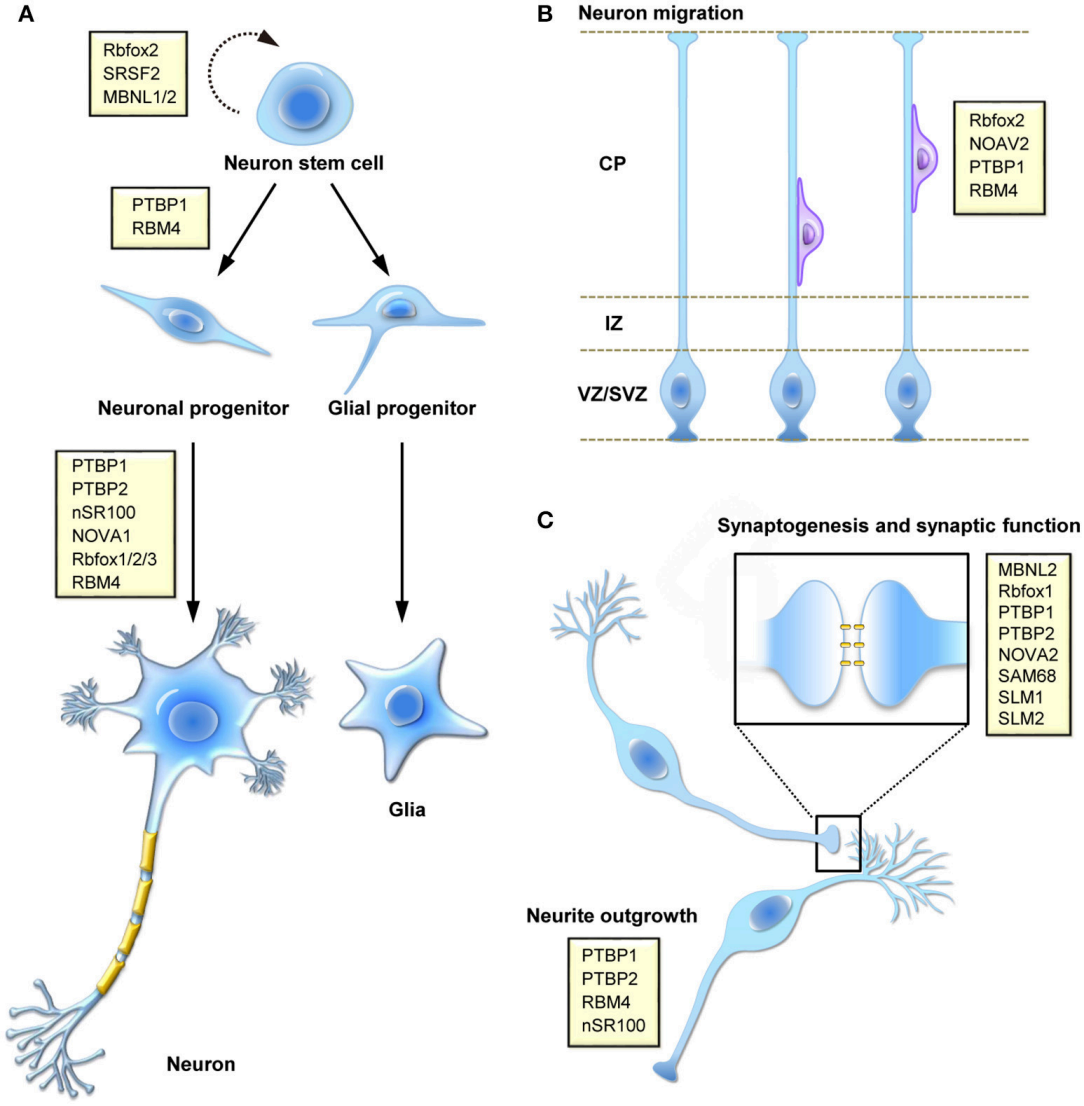
Function of splicing regulatory proteins in the mammalian nervous system. Splicing factors (yellow boxes) participate in a number of different processes during brain development, including **(A)** self-renewing division and fate determination of neural stem cells, and neuronal cell differentiation, **(B)** migration of newly born neuron during corticogenesis, and **(C)** synaptogenesis or neural activity-regulated synaptic function.

Alternative splicing is a crucial step of post-transcriptional gene expression that substantially increases transcriptome diversity and is critical for diverse cellular processes, including cell differentiation and development as well as cell reprogramming and tissue remodeling. Our understanding of the physiological significance and disease implications of alternative splicing has been greatly improved by genetic approaches and RNA deep sequencing. In this review, we focus on alternative splicing in neuronal differentiation from stem/progenitor cells, neuronal migration and functional development of neurons (Figure 1).

### Alternative splicing and its role in development

Approximately 95% of human multi-exon genes undergo alternative splicing of precursor mRNAs (pre-mRNAs) (Pan et al., 2008; Wang et al., 2008). In mammals, alternative splicing involves differential use of intron splice sites or the inclusion/exclusion of exons. Alternatively spliced mRNAs may generate protein isoforms with distinct and perhaps antagonistic functions or with altered stability or subcellular localization (Dredge et al., 2001; Matlin et al., 2005). In addition, alternative splicing may introduce premature termination codons into the resulting mature mRNAs, leading to mRNA downregulation via nonsense-mediated decay (Lareau et al., 2007). Alternative splicing is governed by the interplay between *trans*-acting splicing regulators and *cis*-elements of pre-mRNAs (Matera and Wang, 2014). In general, a splicing activator may enhance splice-site recognition or utilization by the spliceosome, whereas a splicing suppressor may prevent the association of spliceosomal factors with pre-mRNAs or compete off splicing activators. Moreover, alternative splicing is also influenced by transcription rate, histone modifications, and chromatin structure (Kornblihtt et al., 2004, 2013; Luco et al., 2010). Alternative splicing may occur in a tissue- or developmental-specific manner or in response to cellular signals and no doubt plays critical roles in many cellular processes (Nilsen and Graveley, 2010).

Alternative splicing provides a means to differentiate gene expression between cell types during development. Tissue-specific regulation of alternative splicing involves the coordinated actions of splicing factors. Cell type-specific or timely expression of certain splicing regulators is important for precise control of alternative splicing. For example, the RNA-binding protein CUGBP and ETR-3-like factor 1 (CELF1) and muscleblind-like 1 (MBNL1) exhibit switched expression during heart development to regulate splicing of cardiac mRNAs (Kalsotra et al., 2008). Forced expression of embryonic CELF1 or ablation of MBNL1 in the adult mouse heart reverts splicing toward embryonic/early postnatal patterns (Kalsotra et al., 2008). Similarly, switching of splicing regulators also occurs in the developing brain (see below). Thus, temporal control of alternative splicing is critical for fetal-to-adult transitions during development. Coordinated splicing networks contribute substantially to the development of various tissues and organs as well as their physiology.

Splicing abnormalities are linked to human genetic diseases, including brain disorder (Raj and Blencowe, 2015; Vuong et al., 2016). For example, familial dysautonomia is caused by a 5′ splice site mutation of the *IKBKAP* gene (Slaugenhaupt et al., 2001). This mutation reduces *IKBKAP* expression via alternative splicing-coupled nonsense-mediated decay, and hence downregulates a set of cell migration-related genes (Anderson et al., 2001; Yoshida et al., 2015). Gene abnormalities in the splicing factor *RBFOX1* gene have been linked to autism spectrum disorder and additional neuromuscular abnormalities (Barnby et al., 2005; Martin et al., 2007; Conboy, 2017). The associations between splicing defects and human disease have been reviewed extensively elsewhere, and will not be emphasized in this review.

### Experimental insights into the role of alternative splicing in brain development

Emerging new technologies for RNA studies have greatly enhanced our knowledge of alternative splicing in development. Capture of specific mRNA ribonucleoproteins followed by high-throughput sequencing or splicing microarrays has identified dynamic alternative splicing programs during cell differentiation or development and also revealed the tissue-specific or developmentally regulated RNA-binding landscapes of splicing factors (Rossbach et al., 2014). Use of knockout and transgenic mice has identified the targets and physiological roles of neuronal splicing regulators and revealed how their defects impact brain development and neuronal function (Table 1). Moreover, genetic tagging with a reporter provides a tool for isolating specific cell types for transcriptome comparison (Wang et al., 2011). For example, by using *Tbr2* promoter-driven green fluorescent protein as a tracer, neural progenitor cells (NPCs) can be distinguished from neurons in the developing brain (Zhang et al., 2016). Recently, single-cell profiling techniques enabled the resolution of population heterogeneity and revealed insights into cellular differentiation and development (Darmanis et al., 2015). Computational analysis of deep-sequencing data and annotated databases helped establish the correlation between genetic mutations, splicing variants, and disease (Kircher et al., 2014; Mort et al., 2014). Recently, an unbiased “deep-learning” computational method provided a more powerful link between rare single-nucleotide variations and neurological disorders such as spinal muscular atrophy and autism spectrum disorder (Xiong et al., 2015). Advanced sequencing tools would likely facilitate the detection of cell type- and stimulus-dependent splicing changes and perhaps the identification of previously unrecognized splicing products such as circular RNAs during neuronal development (van Rossum et al., 2016).

**Table 1:** Examples of the function of neuronal splicing regulators in neuronal differentiation and brain development.

**Splicing regulators**	**Targets**	**Knockout/downregulation of splicing regulators**		**References**
		**Target exon**	**Phenotypes**	
nSR100/SRRM4	*Protrudin* (*Zfyve27*)	suppressed inclusion of exon L between exon 8 and 9	impaired neurite outgrowth	Ohnishi et al., 2017
Ptbp1 & 2	*PSD-95* (*Dlg4*)	exon 18 inclusion	impaired development of glutamatergic neurons	Zheng et al., 2012
Ptbp1	*Flna*	included the poison exon	brain specific malformation	Zhang et al., 2016
Ptbp2	*Dnm1*	altered mutually exclusive selection of exons 9a/9b	impaired synaptic function, and caused seizures and behavioral deficits	Li et al., 2014
Nova2	*Dab1*	exon 7bc (9bc) inclusion	impaired radial migration and Purkinje neuron migration	Yano et al., 2010
Rbfox3	*Numb*	repressed Numb exon 12 inclusion	impaired neuronal differentiation	Kim et al., 2013
Rbfox1	*Snap25*	altered mutually exclusive selection of exons 5a/5b	caused seizure	Gehman et al., 2011
SRSF1	*ApoER2*	promote exon 19 inclusion	impaired synapse formation and function	Hinrich et al., 2016
hnRNP H1/H2	*TRF2*	exon 7 (TRF2-S) inclusion	impaired neuronal differentiation.	Grammatikakis et al., 2016
RBM4	*Numb*	increased exon 3 skipping and exon 9 inclusion	impaired neuronal differentiation and neuronal outgrowth	Tarn et al., 2016

*PSD-95: postsynaptic density protein 95*.

*Dnm1: dynamin1*.

*Flna: filamin A*.

*Dab1: disabled homolog-1*.

*hnRNP: heterogeneous nuclear ribonucleoprotein*.

*Snap25: synaptosomal-associated protein 25*.

*ApoER2: apolipoprotein E receptor 2*.

*TRF2: telomeric repeat-binding factor 2*.

### Neuronal differentiation involves coordinated changes in the expression of splicing factors

**Figure 2:**
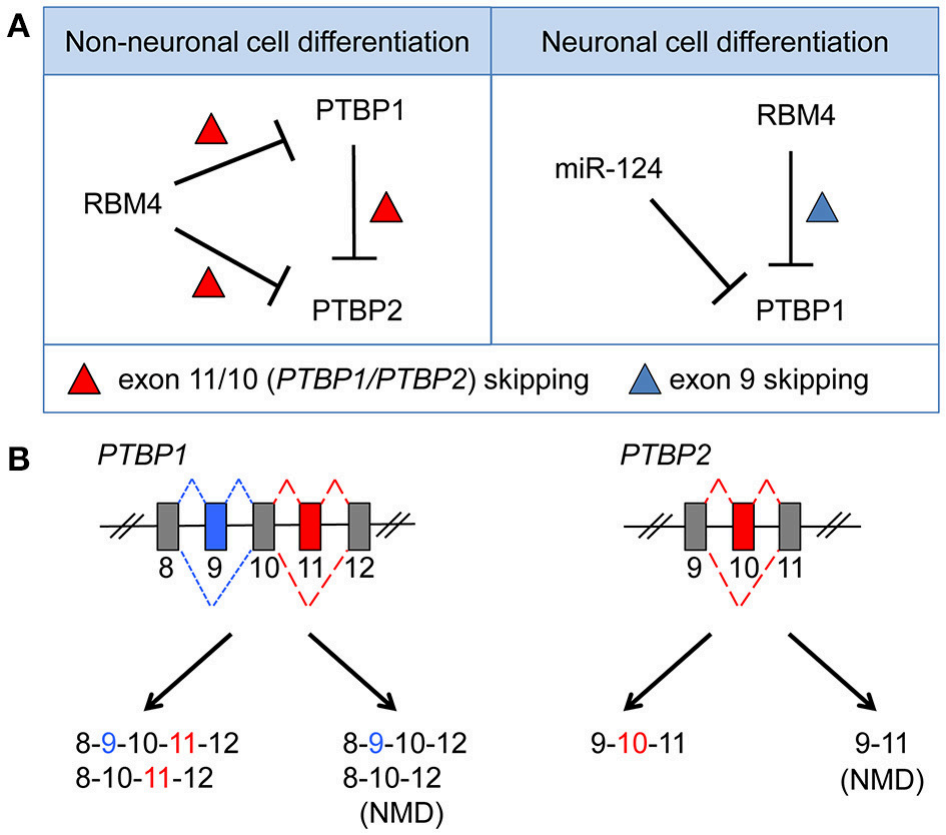
RBM4 regulates PTBP1 expression or splicing activity by modulating exon selection during the differentiation of non-neuronal or neuronal cells. **(A)** RBM4 suppresses the cellular level of both PTBP1 and PTBP2 during non-neuronal cell differentiation via activating exon 11/10 skipping of *PTBP1/PTBP2* mRNAs (Left). PTBP1 also downregulates PTBP2 level by promoting exon 10 skipping of *PTBP2* mRNA (Left). During neuronal differentiation, PTBP1 level is downregulated by miR-124, whereas RBM4-induced exon 9 skipping of *PTBP1* mRNA generates an isoform with reduced splicing activity, which compromises the splicing effect of PTBP1 during neural differentiation (Right). **(B)** Exclusion of exon 11/10 (red box) of *PTBP1/PTBP2* generates splicing isoforms with a premature translation-termination codon, and such isoforms are subjected to degradation via alternative splicing-coupled nonsense-mediated decay. RBM4 promotes exon 9 (blue box) skipping, which is specific to PTBP1.

Genome-wide transcriptome analysis has revealed an exceptionally high level of alternative splicing in the mammalian brain (Yeo et al., 2004). The nervous system adopts alternative splicing for cell differentiation, morphogenesis, the formation of complex neuronal networks, and the establishment/plasticity of delicate synapses (Norris and Calarco, 2012; Zheng and Black, 2013). Splicing regulation may involve some neuron-specific splicing factors and their interplay with ubiquitous factors (Raj and Blencowe, 2015; Vuong et al., 2016). A switch from predominant expression of PTBP1 to its neuronal paralog PTBP2 (nPTB), which occurs during differentiation of progenitor cells into postmitotic neurons, is important for the stem cell-to-neuron transition (Boutz et al., 2007; Vuong et al., 2016). PTBP1 is downregulated by the neuron-specific microRNA miR-124 (Makeyev et al., 2007). Notably, PTBP1 suppresses the inclusion of exon 10 of *PTBP2*, producing an exon 10-skipped mRNA that is susceptible to nonsense-mediated decay (Figure 2). Thus, PTBP1 restricts the level of PTBP2 in non-neuronal cells or NPCs. RBM4 is a ubiquitous RNA-binding protein, but its level is elevated during neuronal differentiation of mouse embryonal carcinoma P19 cells (Tarn et al., 2016). Interestingly, RBM4 acts in the same manner as PTBP1 to suppress exon 11/10 of *PTBP1/PTBP2* in myoblast cells, and it downregulates PTBP1/PTBP2 levels (Lin and Tarn, 2011; Figure 2). However, during neuronal differentiation of mesenchymal stem cells, RBM4 induces the skipping of mammalian-specific exon 9 of *PTBP1*, which produces a functional PTBP1 isoform with compromised splicing activity compared with full-length PTBP1 (Su et al., 2017). Therefore, RBM4 attenuates the activity of PTBP1 in splicing regulation (Su et al., 2017; Figure 2). Notably, *PTBP2* does not contain an exon equivalent of exon 9 of *PTBP1*, so PTBP2 is likely resistant to regulation by RBM4 during stem cell differentiation. On the other hand, the neural-specific SR-related protein of 100 kDa (nSR100/SRRM4) promotes exon 10 inclusion of *PTBP2* and thus maintains PTBP2 level in neurons (Calarco et al., 2009).

PTBP1 and PTBP2 regulate overlapping but distinct repertoires of splicing events. PTBP1 suppresses the splicing of a subset of neural targets to inhibit neuronal differentiation. PTBP2 expression is elevated in differentiating neuronal cells and activates certain neural targets that promote differentiation (Boutz et al., 2007). Nevertheless, PTBP2 is downregulated as cells mature and undergo synaptogenesis. This sequential downregulation of PTBP1 and PTBP2 is important for two transitions of splicing regulation throughout neuronal differentiation and maturation and for functional expression of postsynaptic density protein-95 (PSD-95) via splicing control (Zheng et al., 2012). Both RBM4 and PTBP1 have preference for CU-rich *cis*-elements and hence antagonize each other during splicing regulation; thus, in general, they function oppositely in cell differentiation.

Besides the above, the neuron-specific splicing regulator Nova-1 can negatively autoregulate its own expression by suppressing exon 4 inclusion (Dredge et al., 2005). A study revealed that RBM4 promotes *Nova-1* exon 4 inclusion during differentiation and maturation of brown adipocytes (Lin J. C. et al., 2016), but whether this regulation occurs in neurons is unclear. Moreover, all three Rbfox family members exploit a conserved mechanism of splicing autoregulation to produce a splice isoform with a truncated RNA-recognition motif; this isoform has dominant-negative activity in splicing (Damianov and Black, 2010). The splicing switch of *RBFOX3* from the truncated isoform to the full-length protein occurs in a development-dependent manner, and the latter is necessary for late neuronal differentiation (Kim et al., 2013).

Together, precise timing and level control of splicing regulators is critical for dynamic alternative splicing regulation during cell differentiation and development.

### Alternative splicing in self-renewal and differentiation of stem cells

Alternative splicing also plays a critical role in self-renewal of pluripotent cells as well as in cell-fate determination and reprogramming (Graveley et al., 2011; Ye and Blelloch, 2014). Genome-wide RNA sequencing (RNA-seq) studies have revealed that stem cells and differentiated cells exhibit different splicing profiles (Pritsker et al., 2005). Fine-tuning the expression of several stemness-related transcription factors such as Oct4, Nanog, Sox2, and Tcf3 is important for pluripotency maintenance (Chen et al., 2008; Kim et al., 2008). In particular, different isoforms of *Tcf3* and *Oct4* influence self-renewal of stem cells (Atlasi et al., 2008; Salomonis et al., 2010). The forkhead box transcription factor FoxP1 plays a hierarchical role in the transcription network of pluripotency; the switching of its mutually exclusive exons controls pluripotency and reprogramming of embryonic stem cells (Gabut et al., 2011). Several splicing factors modulate alternative splicing in embryonic stem cells and contribute positively (such as Rbfox2 and SRSF2) or negatively (such as MBNL1/2) to maintaining the stem cell splicing program (Ye and Blelloch, 2014). Thus, alternative splicing plays a critical role in the decision between stem cell self-renewal and differentiation.

Alternative splicing modulates the activity of certain histone modification enzymes in neuronal cells and hence influences the epigenetic status (Fiszbein and Kornblihtt, 2016). The histone methyltransferase G9a is a suppressor of pluripotency-related genes (Kellner and Kikyo, 2010). During neuronal differentiation of neuroblastoma neuro-2a cells, alternative exon inclusion of G9a promotes its nuclear localization and hence increases the dimethylation of histone 3 lysine 9 (H3K9me2). Thus, the regulation of G9a alternative splicing is necessary for efficient neuronal differentiation (Fiszbein et al., 2016). More intriguingly, alternative splicing also modulates the activity of the demethylase LSD1 (Laurent et al., 2015). Therefore, the balanced methylation of H3K9 is likely important for regulating gene expression profiles during neuronal differentiation.

### Alternative splicing in differentiation of neuronal stem/progenitor cells

Transcriptome profiling demonstrated the dynamic nature of alternative splicing events in different cell types, brain regions, and developmental stages (Johnson et al., 2009; Zhang et al., 2014; Yan et al., 2015). RNA-seq analysis of purified NPCs and differentiating neurons in the mouse cortex revealed an alternative splicing switch for a set of neuron-specific exons during differentiation (Zhang et al., 2016). Analysis of human cerebral organoids and fetal neocortex also revealed different splicing patterns in intermediate progenitor cells, redial glial cells, immature neurons, and neurons during cortical development (Camp et al., 2015; Zhang et al., 2016). Therefore, splicing regulation establishes cell type- and stage-specific gene expression profiles during neurogenesis and brain development, which rely on proper expression and function of splicing regulators (Raj and Blencowe, 2015; Vuong et al., 2016; Baralle and Giudice, 2017).

Among neuronal splicing regulators, PTBP1 is exclusively expressed in embryonic stem cells and NPCs, whereas PTBP2 and Rbfox proteins are mainly expressed in neurons. A recent report showed that PTBP1 and Rbfox antagonistically modulate neuronal fate via their roles in regulating alternative exon selection (Zhang et al., 2016). Rbfox switches the centrosomal isoform of *Ninein* to the non-centrosomal form as a result of alternative splicing and hence influences centriolar dynamics and promotes NPC differentiation. On the other hand, PTBP1 suppresses a premature stop codon-containing exon of *filamin A* (*Flna*) in NPCs and hence maintains apical progenitors. Genetic mutations that generate aberrant *Flna* splice isoforms in NPCs are linked to periventricular nodular heterotopia, a neuronal migration disorder. Thus, a better understanding of the mechanisms of neuronal alternative splicing may provide plausible treatment strategies for neuronal disorders.

The Notch receptors play a critical role in fate decisions of various stem/progenitor cells, and Numb is a critical effector of Notch signaling. Alternative splicing of exons 3 and 9 of *Numb* generate four different isoforms, which differentially modulate Notch activity. The detail of how alternative splicing of Numb modulates cell differentiation is not completely known. Rbfox3 can regulate alternative splicing of *Numb*, and Rbfox3 depletion impairs neurogenesis in the hippocampal dentate gyrus (Kim et al., 2013; Lin Y. S. et al., 2016). Our recent study showed that RBM4 determines the selection of two alternative exons, and its overexpression preferentially produces a Numb isoform with the highest potential to promote Mash1 expression and subsequent differentiation of neuronal progenitor cells. Moreover, additional splicing regulators of *Numb* have been implicated in either cancer progression or tumor suppression (Bechara et al., 2013; Zong et al., 2014). Thus, it is conceivable that fine-tuning the expression of Numb isoforms during fate decision of neuronal progenitor cells may constitute a combinatorial effect of multiple splicing regulators.

### Different alternative splicing patterns in neurons and glia

Brain tissues comprise a variety of cell types including neural precursor cells, neurons, and various subtypes of neuroglia. Tantalizing issues remain as to whether and how alternative splicing influences neural fate determination and which splicing regulators are involved (Raj and Blencowe, 2015). Expression of specific alternatively spliced isoforms in distinct neurons has been reported in *Caenorhabditis elegans* and *Drosophila* (Lah et al., 2014; Norris et al., 2014). For example, UNC75 and EXC7 (respective homologs of mammalian CELF and Hu/ELAV) differentially modulate alternative splicing of *unc-16* in GABAergic motor neurons and cholinergic motor neurons (Norris et al., 2014). The energy requirement of different types of brain cells varies; the oxidative and glycolytic pathways predominate in neurons and astrocytes, respectively (Magistretti and Allaman, 2015). Transcriptome profiling has revealed distinct *pyruvate kinase M* (*PKM*) splice isoforms, i.e., *PKM1* and *PKM2* in neurons and glial cells, respectively (Zhang et al., 2014). The *PKM1* and *PKM2* isoforms result from mutually exclusive exon selection. Selective expression of PKM isoforms is also critical for regulating glucose metabolism in muscle and cancer (Christofk et al., 2008). Gradual switching of embryonic PKM2 to adult PKM1 occurs during mouse brain development and during neuronal differentiation of human mesenchymal stem cells (Su et al., 2017). RBM4 antagonizes PTBP1 activity and hence promotes the PKM2-to-PKM1 switch. Overexpression of RBM4 or PKM1 increases oxygen consumption and accordingly facilitates neuronal differentiation. These results support the high energy demand of neurons. Because neuroenergetics is dynamic and changes in response to neuronal activity such as glutamatergic stimulation and hypoxia (Bélanger et al., 2011), whether the expression of the splice isoforms of certain synthetic enzymes, including PKMs, is coordinately changed remains to be investigated. PKM is involved not only in cell metabolism but also in the modulation of gene expression. PKM2 acts coordinately with β-catenin during gene activation underlying the epithelial-to-mesenchymal transition and thus promotes cell proliferation and tumorigenesis (Yang et al., 2011). A recent report demonstrated that the RNA binding protein Quaking maintains neural stem cell functions during early brain development by preventing the PKM2 switch to PKM1 (Hayakawa-Yano et al., 2017).

### Alternative splicing in neuronal migration and brain development

The mammalian cerebral cortex has a highly organized six-layered structure consisting of a variety of neuron subtypes (Molyneaux et al., 2007). Positioning of newborn neurons that originate from the ventricular zone and subventricular zone in the embryonic cortical plate occurs in a birth date-dependent “inside-out” manner (Cooper, 2008; Gao and Godbout, 2013). Several signaling cascades regulate neuronal migration in the cortical plate, including the Reelin-Disabled homolog 1 (Dab1) pathway (Franco et al., 2011; Gao and Godbout, 2013). Upon binding to the very low density lipoprotein receptor (VLDLR) or apolipoprotein E receptor 2 (ApoER2), Reelin induces differential phosphorylation of the cytosolic adaptor protein Dab1 and elicits subsequent downstream events that link Dab1 to the control of neuronal migration. *Reeler* mutant mice and mice with spontaneous or targeted mutations of *Dab1* or either of the receptors exhibit similar phenotypes characterized by ataxia, tremors, and a reeling gait (D'Arcangelo et al., 1995; Howell et al., 1997; Sheldon et al., 1997; Trommsdorff et al., 1999). Differential exon selection of *Dab1* occurs during brain development, resulting in multiple splice isoforms (Gao et al., 2012). Nova2 suppresses the inclusion of mouse *Dab1* exon 9b/c (Yano et al., 2010). Nova2 knockout causes neuronal migration defects in both the cerebral cortex and cerebellum due to increasing aberrant exon 9 b/c-containing *Dab1*. Differential selection of exons 7 and 8 of *Dab1* is also intriguing because these two exons encode a domain containing critical tyrosines that are targets of Reelin-mediated phosphorylation. Moreover, ApoER2 also undergoes alternative splicing. The exon 19-containing domain of ApoER2 is important for synapse formation and function via its interaction with PSD-95 (Beffert et al., 2005; Hinrich et al., 2016). Exon 19 inclusion is reduced in the brain of Alzheimer's patients. It has been shown that SRSF1 inhibits exon 19 inclusion of *ApoER2* and that blocking SRSF1-binding sites using an antisense oligonucleotide has therapeutic potential (Hinrich et al., 2016). Reelin signaling also plays a role in dendritic spine formation and modulates synaptic plasticity in the developing and adult brain (D'Arcangelo, 2014). Therefore, imbalance of splicing factors likely affects neuronal migration and cortical lamination.

### Alternative splicing in neurologic functions

Alternative splicing also regulates neurologic functions such as axon guidance and synaptogenesis. A number of neuronal mRNAs undergo alternative exon selection to generate isoforms in response to neuronal stimulation. Synaptic activity promotes exon 19 inclusion of ApoER2, which then binds Reelin and enhances long-term potentiation (Beffert et al., 2005). Moreover, alternative splicing of the synaptic cell-adhesion molecules neurexins and neuroligins generates multiple isoforms, and interactions between the various isoforms modify their activity toward glutamatergic and GABA-mediated synaptogenesis. Therefore, alternative splicing can shape the strength and functions of synapses. PTBP2 and Sam68 are involved in splicing regulation of neurexins (Resnick et al., 2008; Iijima et al., 2011). Notably, Sam68 activity is regulated by depolarization-induced calcium/calmodulin-dependent kinase IV, indicating that neuronal activity controls the diversity of neurexins via splicing regulation and hence influences synaptic functions (Iijima et al., 2011). Moreover, alternative splicing also regulates the dynamics of neuronal transcriptomes. In pilocarpine-stimulated neurons, exclusion of a cryptic “poison” exon of the sodium channel *Scn9a* mRNA increases the SCN9A level (Eom et al., 2013). A more recent report revealed that neurons can rapidly regulate the expression of several dendritic mRNAs by removing introns that are retained in existing transcripts stored in the nucleus (Mauger et al., 2016). Thus, rapid and signal-responsive splicing regulation is critical for neurological functions.

### Perspectives

The combination of various genetic tools and RNA-seq has advanced our knowledge of the impact of alternative splicing on neural development and function. Recently, the use of cell-surface or genetically engineered fluorescent protein markers and fluorescence-activated cell sorting has enabled the isolation of stem/progenitor cells and specific neuronal types (Zhang et al., 2016). Using Cre recombinase-expressing mouse lines, one can manipulate the temporal expression of a splicing regulator or wild-type or disease-related mutant in specific types of neurons and investigate changes in the transcriptome or splicing patterns or isolate target mRNA ribonucleoproteins (Möröy and Heyd, 2007). Single-cell RNA-seq has begun to clarify cell-to-cell transcriptome variability. Since mammalian brains comprise complex and diverse neuronal cell types, to decipher alternative splicing patterns at the single-neuron level still remains challenged. More recently, a single-cell topological data analysis revealed time-series gene expression changes of individual cells throughout murine embryonic stem cell differentiation into motor neurons (Rizvi et al., 2017). With the aid of new technologies, future investigations will paint a more comprehensive picture and define the dynamic scope of how splicing programming determines stem/progenitor cell fate determination and differentiation into the various brain cell types as well as neural circuit development. Emerging in situ sequencing and single-cell fluorescence in situ hybridization strategies (Liu and Trapnell, 2016) may allow revealing topological changes of alternative splicing in a brain network and perhaps unveiling pathological mechanisms at the single-cell level.

## Author contributions

C-HS, DD, and W-YT: Jointly wrote this review; W-YT: Defined the scope of the review and edited the draft. All authors read and approved the final manuscript.

### Conflict of interest statement

The authors declare that the research was conducted in the absence of any commercial or financial relationships that could be construed as a potential conflict of interest.
